# Human umbilical cord-derived mesenchymal stem cells not only ameliorate blood glucose but also protect vascular endothelium from diabetic damage through a paracrine mechanism mediated by MAPK/ERK signaling

**DOI:** 10.1186/s13287-022-02927-8

**Published:** 2022-06-17

**Authors:** Yi Liu, Jingan Chen, Haowei Liang, Yueqin Cai, Xinyue Li, Li Yan, Li Zhou, Letian Shan, Hui Wang

**Affiliations:** 1 School of Pharmaceutical Sciences, Zhejiang Chinese Medical University, Hangzhou, China; 2 The First Affiliated Hospital, Zhejiang Chinese Medical University, Hangzhou, China; 3 Cell Resource Bank and Integrated Cell Preparation Center of Xiaoshan District, Hangzhou Regional Cell Preparation Center (Shangyu Biotechnology Co., Ltd), Hangzhou, China

**Keywords:** Human umbilical cord-derived mesenchymal stem cells, Diabetic vascular complications, Endothelial damage, Paracrine, MAPK/ERK signaling

## Abstract

**Background:**

Endothelial damage is an initial step of macro- and micro-vasculature dysfunctions in diabetic patients, accounting for a high incidence of diabetic vascular complications, such as atherosclerosis, nephropathy, retinopathy, and neuropathy. However, clinic lacks effective therapeutics targeting diabetic vascular complications. In field of regenerative medicine, mesenchymal stem cells, such as human umbilical cord-derived MSCs (hucMSCs), have great potential in treating tissue damage.

**Methods:**

To determine whether hucMSCs infusion could repair diabetic vascular endothelial damage and how it works, this study conducted in vivo experiment on streptozotocin-induced diabetic rat model to test body weight, fasting blood glucose (FBG), serum ICAM-1 and VCAM-1 levels, histopathology and immunohistochemical staining of aorta segments. In vitro experiment was further conducted to determine the effects of hucMSCs on diabetic vascular endothelial damage, applying assays of resazurin staining, MTT cell viability, wound healing, transwell migration, and matrigel tube formation on human umbilical vein endothelial cells (HUVECs). RNA sequencing (RNAseq) and molecular experiment were conducted to clarify the mechanism of hucMSCs.

**Results:**

The in vivo data revealed that hucMSCs partially restore the alterations of body weight, FBG, serum ICAM-1 and VCAM-1 levels, histopathology of aorta and reversed the abnormal phosphorylation of ERK in diabetic rats. By using the conditioned medium of hucMSCs (MSC-CM), the in vitro data revealed that hucMSCs improved cell viability, wound healing, migration and angiogenesis of the high glucose-damaged HUVECs through a paracrine action mode, and the altered gene expressions of *IL-6, TNF-α*, *ICAM-1*, *VCAM-1*, *BAX*, *P16*, *P53* and *ET-1* were significantly restored by MSC-CM. RNAseq incorporated with real-time PCR and Western blot results clarified that high glucose activated MAPK/ERK signaling in HUVECs, while MSC-CM reversed the abnormal phosphorylation of ERK and overexpressions of *MKNK2*, *ERBB3*, *MYC* and *DUSP5* in MAPK/ERK signaling pathway.

**Conclusions:**

HucMSCs not only ameliorated blood glucose but also protected vascular endothelium from diabetic damage, in which MAPK/ERK signaling mediated its molecular mechanism of paracrine action. Our findings provided novel knowledge of hucMSCs in the treatment of diabetes and suggested a prospective strategy for the clinical treatment of diabetic vascular complications.

**Supplementary Information:**

The online version contains supplementary material available at 10.1186/s13287-022-02927-8.

## Background

Diabetes mellitus is the fastest growing metabolic disorder worldwide, estimated to affect 463 million people in 2019 and projected to affect approximately 693 million by 2045 [1]. Vascular problem, including macrovascular disorders (atherosclerosis and heart disease) and microvascular disorders (nephropathy, retinopathy and neuropathy), is one of the most common complications caused by diabetes mellitus, leading to high risk of cardiovascular diseases, such as heart failure, stroke, venous and arterial thrombosis, cardiomyopathy, and visual impairment [2, 3]. Endothelial damage is a major cause and the earliest event of most vascular complications [3, 4]. As the major cell type of endothelium, endothelial cells play a crucial role in regulating the vascular structure and function by releasing vasoactive factors such as prostacyclin (PGI 2), reactive oxygen species (ROS), endothelin-1 (ET-1), nitric oxide (NO), and angiotensin II (Ang II) [5, 6]. After long exposure to a hyperglycemic environment, endothelial cells increasingly secrete vasoconstrictive and inflammatory factors (e.g., TNF-α, Ang II, ROS, ET-1) and reduce NO production and high-density lipoprotein (HDL) uptake, resulting in vulnerable vasculature, enhanced oxidative stress, impaired endothelial repair, and a pro-atherogenic state [3, 7]. Moreover, hyperglycemia severely inhibits endothelial cell viability, elevates endothelial permeability, and accelerates endothelial cell apoptosis and aging [8]. Thus, hyperglycemic damage of endothelial cells is the causative factor of diabetic vascular complications, attracting increasing attentions and requiring targeted therapeutics [9]. To date, there is little medication targeting diabetic vascular complications, and the commonly used drugs, such as biguanides (metformin), sulfonylureas, thiazolidinediones (glitazones), meglitinides (glinides), and alpha-glucosidase inhibitors, only target blood glucose homeostasis to indirectly repair the vascular damage [10, 11]. Even so, those drugs have side effects in clinic, which are not satisfied for the treatment of diabetic vascular complications [10, 12]. Hence, new approaches are needed to specifically restore the vascular complications of diabetes mellitus.

Currently, mesenchymal stem cells (MSCs)-based cell therapy has been recognized as an effective approach for many diseases, such as diabetes mellitus and its complications [13, 14]. With the homing and paracrine capacities, MSCs enable to migrate to the injured tissue site and release extracellular vesicles, growth factors, chemokines and cytokines to modulate the immune response and repair the injured tissue [15, 16]. For instance, MSCs secrete growth factors (bFGF, IGF-1, HGF and VEGF, etc.) to stimulate cell proliferation and tissue reorganization, secrete anti-inflammatory factors (IL-10 and IL1-RA) to suppress local inflammation, and secrete exosomes and extracellular RNAs to trigger intercellular communication and regulation [17–19]. Intravenous injection of MSCs into diabetic rats exhibited efficacy in reducing body weights, lowering blood glucose levels, and improving glucose tolerance through increasing insulin secretion, repairing islet damage and replenishing β cell function [20, 21]. Clinical studies showed that diabetic patients infused with MSCs exhibited significantly decreased postprandial glucose and HbA1c levels [22]. Moreover, MSCs possesses therapeutic potential in treating diabetic complications, such as retinopathy, nephropathy, and foot ulcer, by improving angiogenesis, decreasing oxidative stress damage and macrophage infiltration, and accelerating tissue repair and regeneration [23–25]. Therefore, MSCs therapy is a promising approach for treating diabetes mellitus and its complications. However, whether MSCs can be used to restore diabetic vascular damage, remains unclear.

Considering the tissue-repair and pro-angiogenesis capacities of MSCs, we proposed a hypothesis that MSCs could restore diabetes-induced damage of vascular endothelium through paracrine action. To verify this, we employed human umbilical cord-derived mesenchymal stem cells (hucMSCs) to treat diabetic model of rats as well as cellular model of human endothelial cells (HUVECs), and the efficacy and paracrine mechanism of hucMSCs on diabetic endothelial damage were explored. The findings of this study may provide new knowledge of hucMSCs in treating diabetic vascular complication, indicating hucMSCs as an ideal source of cell therapy in clinic.

## Materials and methods

### Chemicals and reagents

Minimum essential medium-alpha modification (α-MEM) with Glutamax™-1 was purchased from Gibco BRL (NY, USA). hucMSCs were obtained from Cell Resource Bank and Integrated Cell Preparation Center of Xiaoshan (Hangzhou, China). HUVECs were purchased from the Chinese Academy of Sciences (Beijing, China). Dulbecco’s modified Eagle’s medium (DMEM) was purchased from Zhejiang Senrui Biotechnology Co., Ltd. Trypsin (0.25%) was purchased from Biosharp (Beijing, China). Fetal bovine serum (FBS) was purchased from Gibco BRL (NY, USA). Resazurin was purchased from Shanghai Yuanye Biotechnology Co., Ltd. 3-(4,5-dimethylthiazol-2-yl)-2,5-diphenyltetrazolium bromide (MTT) was purchased from Sigma-Aldrich, USA. Cell culture plates were purchased from ThermoFisher Scientific Inc. (Waltham, MA, USA). Transwell chambers and Matrigel® Growth Factor Reduced Basement were purchased from Corning (NY, USA). ELISA kits were purchased from MeiMian Bio (Wuhan, China). TRIzol reagent kit and TaqMan cDNA Synthesis kit was obtained from Thermo Fisher Scientific, Inc. (MA, USA). The miScript SYBR Green PCR kit was obtained from Qiagen (Dusseldorf, Germany). BCA Protein Assay Kit was purchased from Beyotime Institute of Bio (JiangSu, China). The antibodies (anti-ERK1/2, anti-phospho-ERK1/2, anti-histone H3 (HH3) and horseradish peroxidase–conjugated antibodies) were purchased from Cell Signaling Technology Inc. (MA, USA), and anti-GAPDH were purchased from Bioker Baoke Biotechnology Co., Ltd (Hangzhou, China). Streptozotocin (STZ) was obtained from Sigma (Saint Louis, USA). Citrate buffer was purchased from Solarbio, (Beijing, China). Triton X-100 was purchased from Invitrogen, (CA, USA).

### Flow cytometry identification of hucMSCs

Flow cytometry was used to identify the hucMSCs. The cells were cultured in α-MEM containing 10% FBS at 37 °C with 5% CO_2_ and were collected with cell density of 1 × 10^9^ cells per tube. Respectively, CD34, CD45, HLA-DR, CD473, CD90, and CD105 antibodies were added for incubation at 4 °C in dark for 30 min, and the positive rate of those surface markers was analyzed by flow cytometry (BD Accuri C6, NY, USA).

### Animal experimentation

Male SD rats (250–280 g, 8 weeks old) were purchased from Shanghai SLAC Laboratory Animal Co., Ltd, China and were housed under standard environmental conditions (22 ± 2 °C, 55–60% relative humidity, and 12 h light/12 h dark cycle) with free access to tap water and food. All rats were kept in SPF animal room for 7 days for proper acclimatization. Great care was taken to minimize their suffering and this study was approved by the Animal Ethics Committee of Zhejiang Chinese Medical University (Animal Ethics No: 11410).

Thirty two rats were prepared as follows: twenty four rats received tail-vein injection of freshly prepared STZ in citrate buffer (dissolved in 0.1 mM citrate buffer, pH 4.2–4.5) at a dose of 50 mg/kg, and eight rats received an equal volume of citric buffer as control. After 7 days, the STZ-injected rats with blood glucose level ≥ 16.1 mM were considered as diabetic rats. Control and diabetic rats were randomly divided into four groups: (i) control group (n = 8), (ii) model (diabetes mellitus) group (*n* = 8), (iii), low dose hucMSCs-treated (MSC-L, 5 × 10^6^ cells per rat) group (n = 8), and (iv) high dose hucMSCs-treated (MSC-H, 1 × 10^7^ cells per rat) group (n = 8). Among them, intravenous injections of hucMSCs into rat tail in the MSC-L and MSC-H groups were performed once a week for 4 weeks. The body weight and fasting blood glucose (FBG) were measured at each week. After the 4-week treatment, all rats were euthanized to collected their thoracic aorta and serum for further experiments.

### Biochemical analysis

The rat ICAM-1 and VCAM-1 were detected by using the commercially available ELISA kits. The optical density was measured at 450 nm using a microplate reader (Multiskan™ FC, ThermoFisher Scientific Inc., Waltham, MA, USA). The levels of ICAM-1 and VCAM-1 were quantified based on the standard curves that were constructed by the CurveExpert software (Version 1.4, Chattanooga, USA). Three replicated wells from each sample were assayed.

### Histopathological observation and immunohistochemical staining

Thoracic aorta samples were fixed with 4% paraformaldehyde for 24 h and then washed with tap water and gradient ethanol (75%-85%-95%-100%) for dehydration. Afterwards, the samples were cleaned in xylene and subsequently embedded in paraffin. Each segment was sectioned into 5 µm thickness with a slicing microtome. In histopathological analysis, the sections collected on glass slides were stained with haematoxylin and eosin (HE) and then imaged using a microscope (Nikon Eclipse 80i, Tokyo, Japan). In immunohistochemical staining, the protein expressions of p-ERK and Histone H3 were detected in thoracic aorta samples. To repair antigens, the sections were treated with 0.01 mol/l citrate buffer (pH 6.0, Solarbio, Beijing, China) at 60 °C for 4 h, washed three times with PBS, incubated with 0.1% Triton X-100 (HFH10, Invitrogen, USA) for 10 min, washed three times with PBS and then sealed with goat serum for 30 min. Followed by the overnight incubation at 4 °C with the primary antibody of p-ERK and Histone H3 (1: 400 dilution). All sections were treated with Horseradish peroxidase-conjugated secondary antibody (PV-9002 for p-ERK and PV-9001 for Histone H3) for 20 min and then with 3,3′-diaminobenzidine (DAB) substrate chromogen for 8 min. Finally, all sections were imaged using a microscope (Nikon Eclipse 80i, Tokyo, Japan) and the immunoreactivity of p-ERK and Histone H3 was mean optical density by using Image-Pro Plus 6.0 software (Media Cybernetics, Bethesda, MD, USA).

### Conditioned medium preparation of hucMSCs and HUVEC

HucMSCs were seeded into 10 cm dishes and grown to 80% ~ 90% confluence in α-MEM. After the removal of supernatant, DMEM was added into the dishes for another 48 h. The replaced supernatant was collected as the conditioned medium of hucMSCs (MSC-CM). The conditioned medium of HUVEC (EC-CM) was prepared in DMEM as above and was used as parallel control for MSC-CM, so that the medium would not affect the outcome.

### Cellular experimentation

To investigate the effects of hucMSCs on HUVECs, two treatment methods were applied in this study, including the direct treatment of MSC-CM and co-culture treatment of hucMSCs-HUVECs. Initially, HUVECs were cultured in DMEM containing 10% FBS and incubated at 37 °C in a humidified atmosphere containing 5% CO_2_. The expanded HUVECs were divided into three groups as follows: (i) control group in normal medium (25 mM glucose) with EC-CM treatment; (ii) model group in high glucose (50 mM glucose) with EC-CM treatment; and (iii) MSC-CM group in high glucose with MSC-CM treatment. Subsequently, in the direct treatment of MSC-CM, for cellular modeling, the cells were treated with high glucose for 48 h. After the removal of supernatant, EC-CM was added into the control group, and MSC-CM was added into the MSC-CM group for another 48 h. Moreover, in the co-culture experiment of hucMSCs-HUVECs, HUVECs were seeded into the 6-well plate and modeled in high glucose for 48 h, after discarding the supernatant, the chamber soaked for 1 h in advance were placed in the MSC group, hucMSCs (3 × 10^5^ cells/well) was added to the upper layer, and high glucose DMEM medium was added to the lower layer for 48 h.

### Cell viability assay

To evaluate the effect of MSC-CM on the viability of HUVECs, MTT assay and resazurin assay were performed, respectively. In MTT assay, HUVECs in each group were seeded into 96-well plates (3000 cells per well). After the treatment, an aliquot of 50 μl MTT solution was added into each cell for 4 h at 37 °C in the dark. Subsequently, the supernatants were removed and 150 μl DMSO was added into each well and shaken for 10 min. The optical density (OD) values of each well were measured at 490 nm using a microplate photometer (Multiskan™ FC, ThermoFisher Scientific Inc., Waltham, MA, USA). Moreover, in resazurin-based assay, HUVECs (1500 cells per well) were inoculated in 24-well plates. After the treatment, 500 μl PBS with 25 μg/mL resazurin sodium salt (7-hydroxy-3H-phenoxazin-3-one-10-oxide) was added to each well. After incubation with 5% CO2 at 37° C for 4 h. the fluorescence intensity was detected at 560/590 nm using the microplate photometer (Fluoroskan Ascent ™ FL, ThermoFisher Scientific Inc., Waltham, MA, USA).

### Matrigel tube formation assay

Tube formation assay was applied to test the angiogenetic potential of HUVECs. 96-well plates were pre-coated with 50 μl of Matrigel® Growth Factor Reduced Basement and polymerized for 30 min at 37 °C. HUVECs were seeded into each well at a density of 4 × 10^4^ cells per well and divided into control, model (HG), and MSC-CM groups. The control group was treated with normal DMEM (25 mM glucose) for 48 h and EC-CM for another 48 h, the model (HG) group was treated with high-glucose DMEM (50 mM glucose) for 48 h and EC-CM for another 48 h, and the MSC-CM group was treated with MSC-CM for 48 h after the modeling as the model group. Afterwards, the tube network formation was observed under an inverted light microscope (Carl Zeiss, Gottingen, Germany), and statistical analysis was conducted Image-J software (Version 1.49, National Institutes of Health, Bethesda, USA).

### Wound healing assay

Wound healing assay was applied to test the horizontal migration capability of HUVECs. The cells (6000 cells/well) were seeded in 6-well plates and grouped as above. After cell adhesion, a sterile 10 μl pipette tip was used to scrape the cell monolayer to create a rectangular cell-free zone in each well. The remaining cells were washed twice with PBS and cultured for 24 h. The cell migration was observed and photographed using an inverted microscope (Carl Zeiss, Gottingen, Germany), followed by statistical analysis of the cell-free zone by Image-J software (Version 1.49, National Institutes of Health, Bethesda, USA).

### Transwell assay

Transwell assay was applied to test the vertical migration capability of HUVECs. The cells were seeded in the upper chamber of inserts in 24-well plates and grouped as above. Cells that with the intervention were digested with trypase and added to the upper chamber. After 15 h the upper chamber of inserts were gently removed and HUVECs migrated to the bottom of each well were fixed with 4% paraformaldehyde and then stained with haematoxylin and eosin. The number of migrated HUVECs was calculated by counting on five random microscope fields (× 200) using the FLEXACAM C1 microscope (Leica, Wetzlar, Germany).

### RNA sequencing (RNAseq) analysis

To initially explore the molecular action mechanism of hucMSCs on the diabetes-damaged HUVECs, RNAseq was employed and the transcriptome changes of HUVECs were profiled. The total RNA of HUVECs was extracted by Trizol reagent from the control, model, and MSC-CM groups after 48 h treatment, followed by quantification using NanoDrop 2000 spectrophotometer (ThermoFisher Scientific Inc., MA, USA) and quality confirmation using Agilent 2100 Bioanalyzer (Agilent, Waldbronn, Germany). mRNA was obtained from total RNA by using Dynabeads Oligo (dT) (Life Technologies, New York, USA), sheared to fragments of ~ 300 bp by fragmentation buffer, and reverse-transcribed into complementary DNA so as to establish the cDNA library using Illumina TruseqTM RNA sample prep Kit (Illumina, San Diego, California, USA). The sequencing library was qualified by Qubit 2.0 (Life technologies, ThermoFisher Scientific Inc., Waltham, USA) and Agilent 2100 Bioanalyzer (Agilent, Santa Clara, CA, USA), and the DNA sequencing was performed on the Illumina Novaseq 6000 Sequencing System (Illumina, San Diego, California, USA).

For bioinformatic analysis, the RNAseq dataset was analyzed for the gene and transcript levels among control, model, and MSC-CM groups using the DESeq2 software (Version 3.14, MA, USA). The differentially expressed genes (DEGs) among the three groups were obtained based on the threshold of |log2 FC|≥ 0.585 and *P* adjust < 0.05. Then, Kyoto Encyclopedia of Genes and Genomes (KEGG) database (http://www.kegg.jp/) was utilized for pathway enrichment analysis by using based clusterprofile package in R language software (Version 4.0.5, Lucent Technologies, Bell Laboratories, Jasmine mountain, New Jersey, USA). Hypergeometric distribution test was conducted to determine the significance (*P* < 0.05) of enriched KEGG pathway.

### Real-time PCR analysis

To determine the gene expression regarding cell viability, migration, senescence, and tube formation as well as to verify the RNAseq result. Real-time polymerase chain reaction (qPCR) analysis was performed. Total RNA was extracted and reverse‑transcribed into cDNA as above. The running condition for qPCR was set in order of a holding stage for one cycle (30 s at 95 °C), a cycling stage for 40 cycles (5 s at 95 °C and 30 s at 60 °C), and a melting curve stage for one cycle (15 s at 95 °C, 30 s at 60 °C and 15 s at 95 °C). The primer sequences of the targeted genes were shown in Table 1. The final data was exhibited by using the ^ΔΔ^Ct method.

**Table 1:** Primer sequences used for real-time PCR analysis

Gene	Forward primer	Reverse primer
*β-actin*	GTGGACA TCCGCAA AGAC	AAAGGGTGTAACGC AACTA
*IL-6*	CACTGGTCTTTTGGAGTTTGAG	GGACTTTTGTACTCATCTGCAC
*TNF-α*	CAGAGGGAAGAGTTCCCCAG	CCTTGGTCTGGTAGGAGACG
*ICAM-1*	TGATGGGCAGTCAACAGCTA	GGGTAAGGTTCTTGCCCACT
*VCAM-1*	GCTGCTCAGATTGGAGACTCA	CGCTCAGAGGGCTGTCTATC
*P53*	TTCCTGAAAACAACGTTCTGTC	AACCATTGTTCAATATCGTCCG
*P16*	CATGGTGCGCAGGTTCTTG	CGGGATGTGAACCACGAAA
*ET1*	TAGCCAAAAAGACAAGAAGTGC	TTCTTCCTCTCACTAACTGCTG
*BAX*	CGAACTGGACAGTAACATGGAG	CAGTTTGCTGGCAAAGTAGAAA
*DDIT3*	GGAAACAGAGTGGTCATTCCC	CTGCTTGAGCCGTTCATTCTC
*MYC*	CGACGAGACCTTCATCAAAAAC	CTTCTCTGAGACGAGCTTGG
*MKNK2*	CCAGCCGAACTTCAGGGTTT	CGTCCGGGATGTCAATGGG
*DUSP5*	TGTCGTCCTCACCTCGCTA	GGGCTCTCTCACTCTCAATCTTC

### Western blot

The protein expressions of HUVECs and thoracic aortas were determined by using Western blot (WB) analysis. To ensure the repeatability of WB experiments in vitro, HUVECs were grouped and treated as above for three times, and the total proteins of each sample were extracted with a lysis buffer containing PMSF. To ensure the repeatability of WB experiments in vivo, the thoracic aorta samples from three individual rats were obtained in the control, model, MSC-L, and MSC-H groups, respectively. For the protein extraction of thoracic aortas, high temperature sterilization scissors were used to cut part of the rat aortic tissue, which was crushed and placed in a glass homogenizer, adding an appropriate amount of pyrolysis liquid, grinding and cracking on ice for 30 min, 12 000 g, centrifuged at 4 °C for 5 min, and the supernatant was transferred to the pre-cooled centrifuge tube. The protein concentrations of all samples from HUVECs and thoracic aortas were determined by using a BCA Protein Assay Kit. After the protein concentration was determined by BCA method, the sample buffer was boiled and denatured to obtain aortic proteins. The proteins were separated by sodium dodecyl sulfate–polyacrylamide gel electrophoresis (SDS-PAGE) and blotted onto polyvinylidene difluoride membranes. WB was performed using antibodies of ERK1/2, phosphorylated ERK1/2, and GAPDH. The membranes were incubated overnight with the primary antibodies against ERK1/2, phosphorylated ERK1/2, and GAPDH at 4 °C. After thorough washing, the membranes were further incubated with horseradish peroxidase–conjugated secondary antibodies for 2 h. Finally, the blots were displayed by using an enhanced chemiluminescence detection system (Millipore Corp, Bedford, MA, USA).

### Statistical analysis

All statistical analyses were performed using SPSS 22.0 software (SPSS, Chicago, USA) and GraphPad Prism Software (Version 8.4, San Diego, CA, USA). Results were expressed as means ± standard deviation (SD) from at least three independently replicated experiments and were analyzed by one way ANOVA based on least-significant difference (LSD) method. *P*-value < 0.05 was considered statistically significant.

## Results

### Isolation and characterization of hucMSCs

**Fig. 1:**
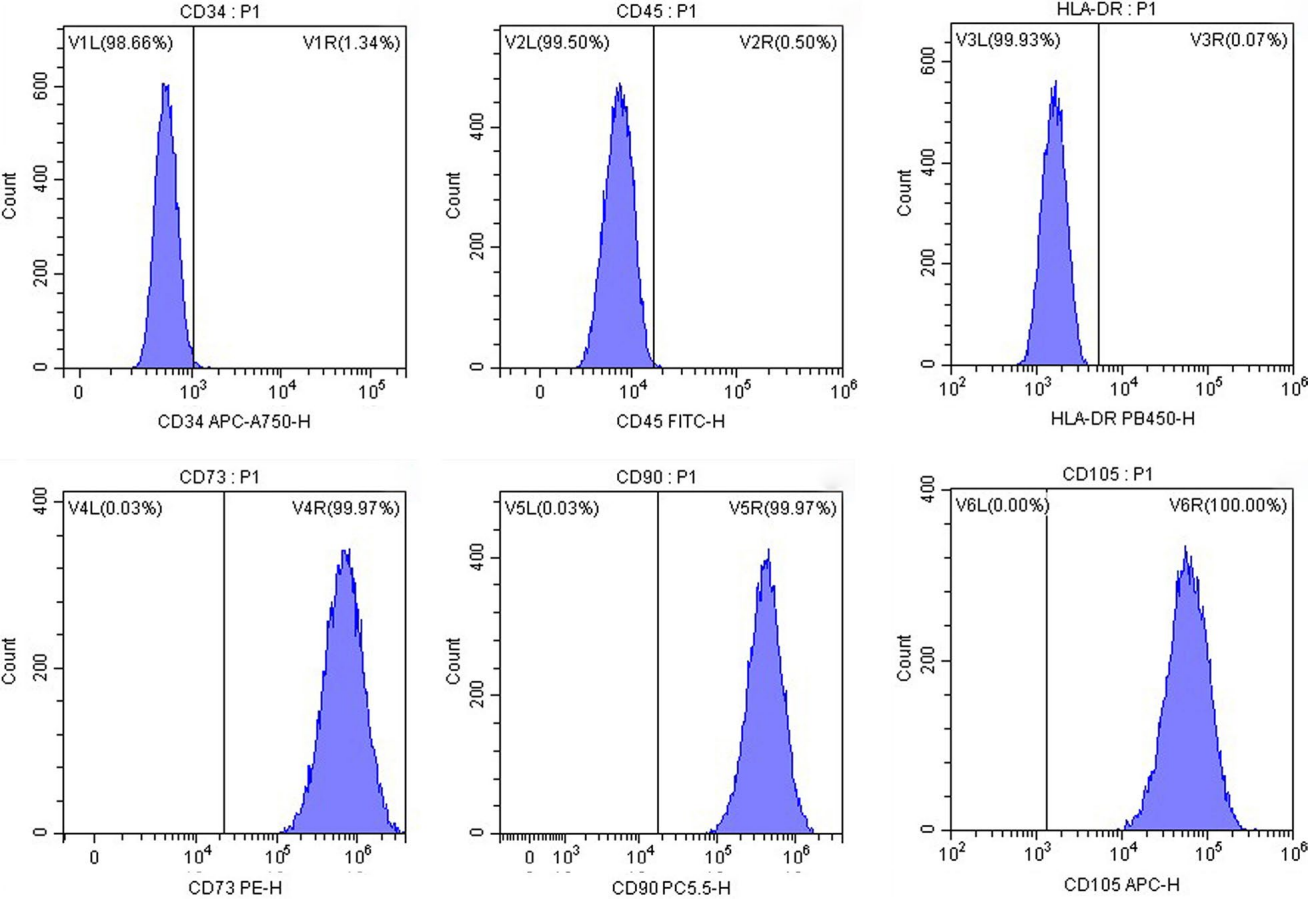
Surface marker expression of hucMSCs measured by flow cytometry analysis

According to the identification criteria of the International Society for Cellular Therapy, MSCs is characterized by high expression (≥ 95%) of positive markers (CD105, CD73, and CD90) and lack expression (≤ 2%) of negative markers (CD45, CD34, and HLA-DR) [26]. In Fig. 1, the flow cytometry result showed high expression of CD73 (99.97%), CD90 (99.97%), and CD105 (100.00%) along with low expression of CD34 (1.34%), CD45 (0.50%) and HLA-DR (0.07%) of our cell samples, confirming the identity of hucMSCs.

### Effect of hucMSCs on biological parameters of diabetic rats

**Fig. 2:**
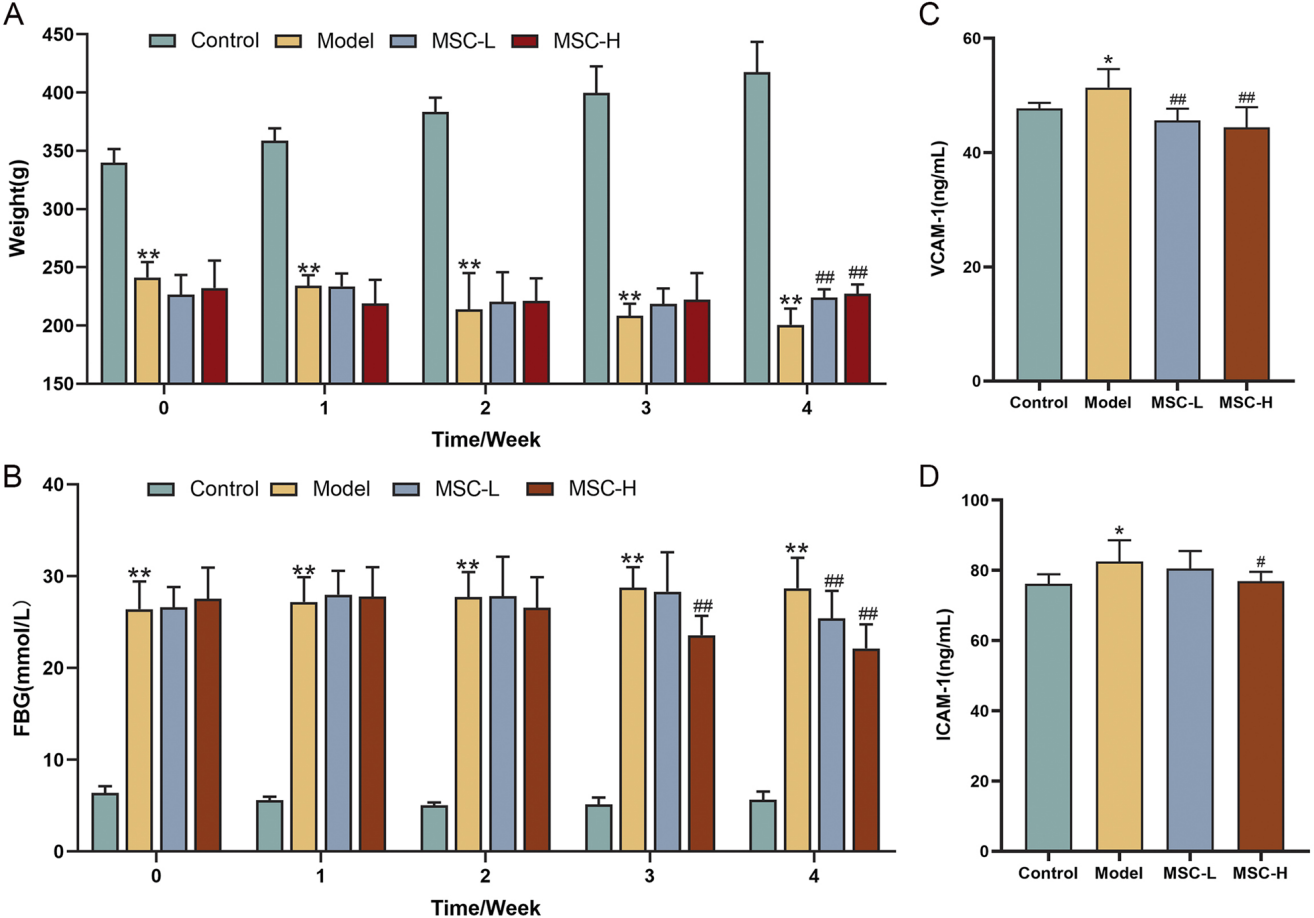
The body weight (**A**), fasting blood glucose (FBG) (**B**), and blood levels of VCAM-1 (**C**) and ICAM-1 (**D**) in the control (*n* = 8), model (*n* = 8), MSC-L (*n* = 8) and MSC-H (*n* = 8) groups. The data at 0 week was obtained before hucMSCs treatment, and the data at 1, 2, 3, 4 week were obtained 7 days after each hucMSCs treatment. Data were mean ± SD. *P < 0.05 and **P < 0.01 versus the control group, ^#^*P* < 0.05 and ^##^*P* < 0.01 versus the model group

To evaluate the therapeutic effect of hucMSCs on blood glucose and endothelial vascular function, the body weight and FBG of diabetic rats were weekly monitored before (0 week) and after the injection of hucMSCs (1, 2, 3, 4 week). The monitoring time point was the day before the next injection. Moreover, the blood levels of ICAM-1 and VCAM-1 were detected after 4-week treatment of hucMSCs (7 days after the final injection). In Fig. 2A, the body weight of normal rats in the control group was continuously increased, while that of diabetic rats in the model group was dramatically decreased (each *P* < 0.01 vs control level). Compared with the model group, the body weight of diabetic rats in both MSC-L and MSC-H groups were significantly increased at the 4th week after hucMSCs injection (each *P* < 0.01 vs model level). In Fig. 2B, FBG of diabetic rats in the model group was dramatically increased as compared with that of the control rats during the 4-week treatment (each *P* < 0.01 vs control level). Compared with the model group, the FBG was significantly decreased in the MSC-L group after 4 weeks and in the MSC-H group after 3 weeks (each *P* < 0.01 vs model level). In Fig. 2C, D, the levels of ICAM-1 and VCAM-1 in the model group were significantly higher than that in the control group (each *P* < 0.05 vs control level), while the levels of VCAM-1 in both MSC-L and MSC-H groups were significantly restored to normal level (each *P* < 0.01 vs model level) and ICAM-1 in the MSC-H group was restored (*P* < 0.05 vs model level).

### Effect of hucMSCs on aorta histopathology of diabetic rats

**Fig. 3:**
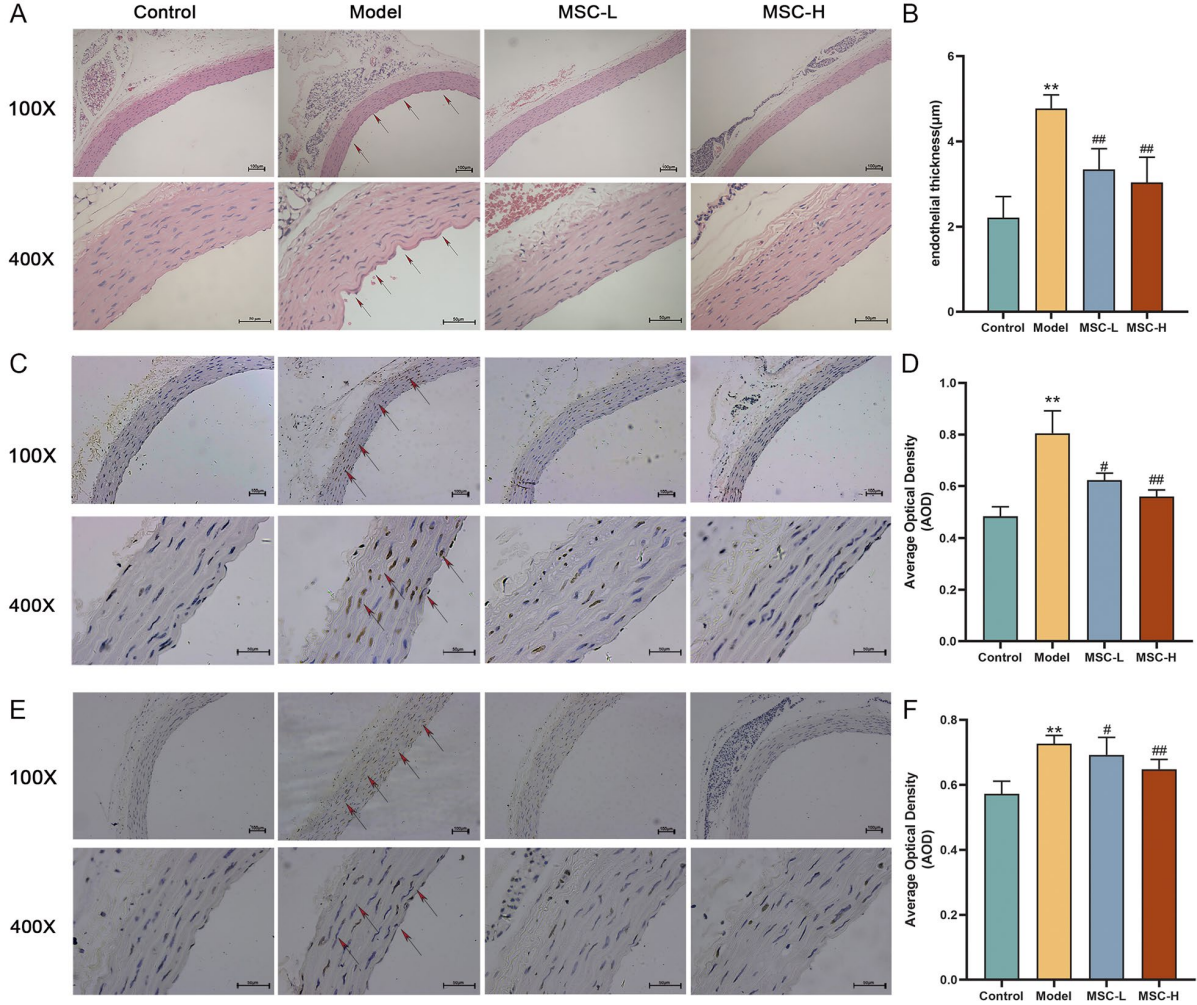
The effect of hucMSCs on diabetes-induced histopathological alterations of aorta endothelium. **A** HE staining of aortic morphology (100 × and 400 ×), and **B** aortic endothelial thickness. **C**, **D** The representative images (100 × and 400 ×) of immunohistochemical staining of p-ERK and the average optical density values of p-ERK. **E** and **F** The representative images (100 × and 400 ×) of immunohistochemical staining of HH3 and the average optical density values of HH3. (data were mean ± SD). **P < 0.01 versus control, ^#^*P* < 0.05 and ^##^*P* < 0.01 versus model

As shown in Fig. 3A, HE staining showed normal phenotype of endothelium of normal rats in the control group, including normal thickness of endothelial layer, smooth and clear boundary of intima, and well-arranged endotheliocytes, while that of model rats was abnormal, including increased thickness of endothelial layer, rough and disordered intima, and absent or irregularly shaped endotheliocytes, indicating diabetes-induced damage of endothelium. After the treatment with hucMSCs at low and high doses, the histological morphology of aorta endothelium was obviously improved, exhibiting smooth and complete intima and restored endotheliocytes. Moreover, Fig. 3B shows that the average endothelial thickness of model rats was significantly higher than that of normal rats (*P* < 0.01 vs control level) and was significantly restored by both MSC-L and MSC-H (each *P* < 0.01 vs model level). The results of immunohistochemistry showed that p-ERK and HH3 positive brown signals in the model group were significantly altered (*P* < 0.01 vs control level), while those in the MSC-L and MSC-H groups were restored (Fig. 3D, F, *P* < 0.05 or *P* < 0.01 vs model level).

### Paracrine effects of hucMSCs on the viability, and migration of HUVECs

**Fig. 4:**
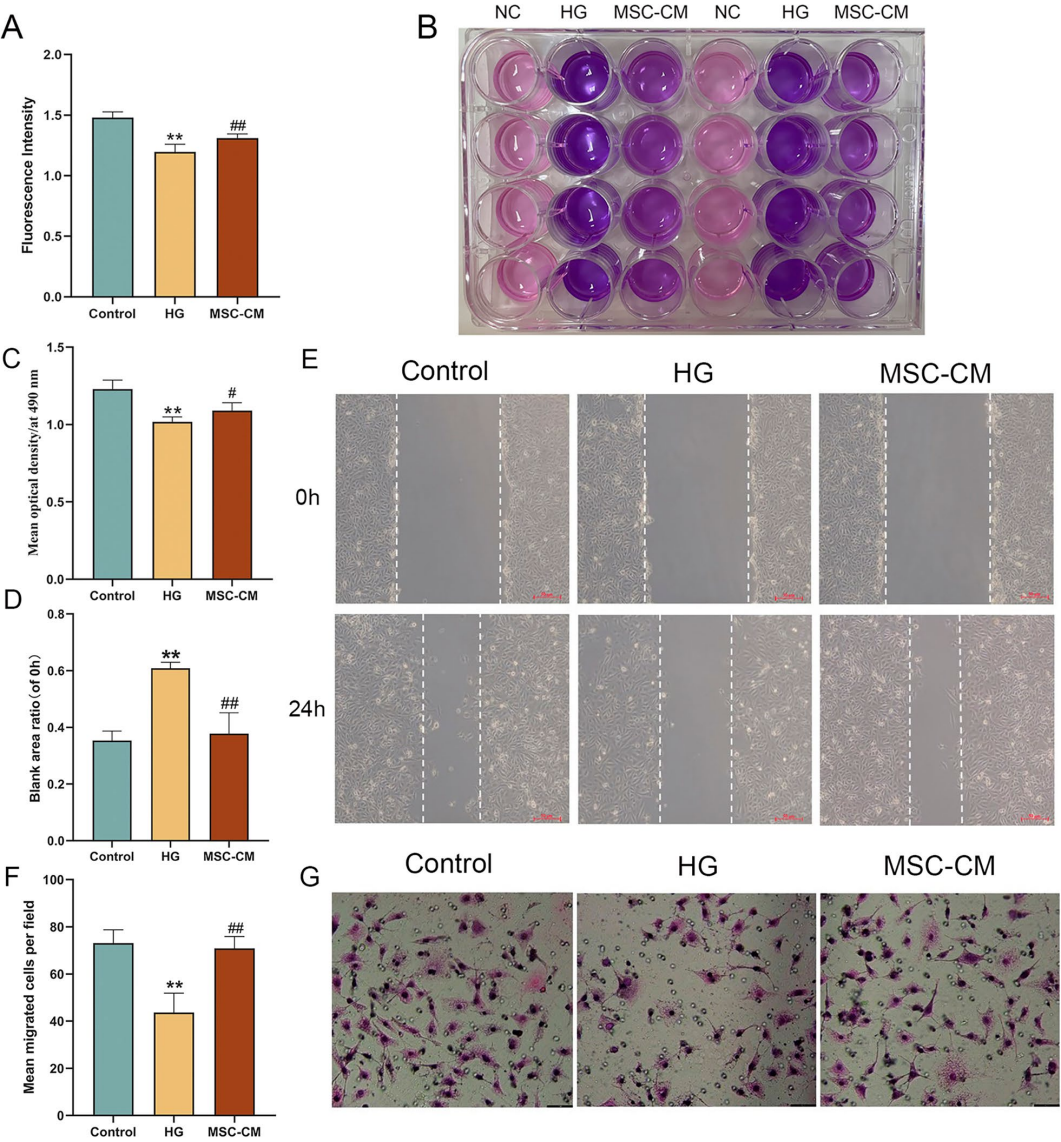
The effects of MSC-CM on the cell viability, wound healing and cell migration of HUVECs in the control, HG and MSC-CM groups. **A**, **B** showed the fluorescence intensity and the representative pictures in the resazurin-based assay. **C** Cell viability detected by MTT method. **D**, **E** showed wound healing rate and representative pictures in the scratch test (scale bar = 50 μm). **F**, **G** showed the number of cell migration and the representative pictures in the transwell experiment, and each group randomly selected four fields for cell counting (scale bar = 50 μm). Data were mean ± SD, **P < 0.01 versus control level, #*P* < 0.05 and ^##^*P* < 0.01 versus model level

To determine the paracrine effects of hucMSCs on HUVECs, MSC-CM was applied and cell viability, wound healing and transwell migration, were performed. In Fig. 4A, B, the fluorescence intensity of the model (HG) group was significantly lower than that of the control group (*P* < 0.01 vs control level), while that in the MSC-CM group was significantly restored to the normal level (*P* < 0.01 vs model level). In Fig. 4C, the MTT optical density of HUVECs in the HG group was significantly decreased (*P* < 0.01 vs control level), while that in the MSC-CM group was significantly restored to normal (*P* < 0.05 vs model level). In Fig. 4D, E, the wound healing of HUVECs in the HG group was significantly inhibited (*P* < 0.01 vs control level), while that in the MSC-CM group was significantly restored to normal (*P* < 0.01 vs model level). In Fig. 4F, G, the transwell migration of HUVECs in the HG group was significantly inhibited (*P* < 0.01 vs control level), while that in the MSC-CM group was significantly restored to normal (*P* < 0.01 vs model level).

### Paracrine effect of hucMSCs on tubule network formation of diabetic HUVECs

**Fig. 5:**
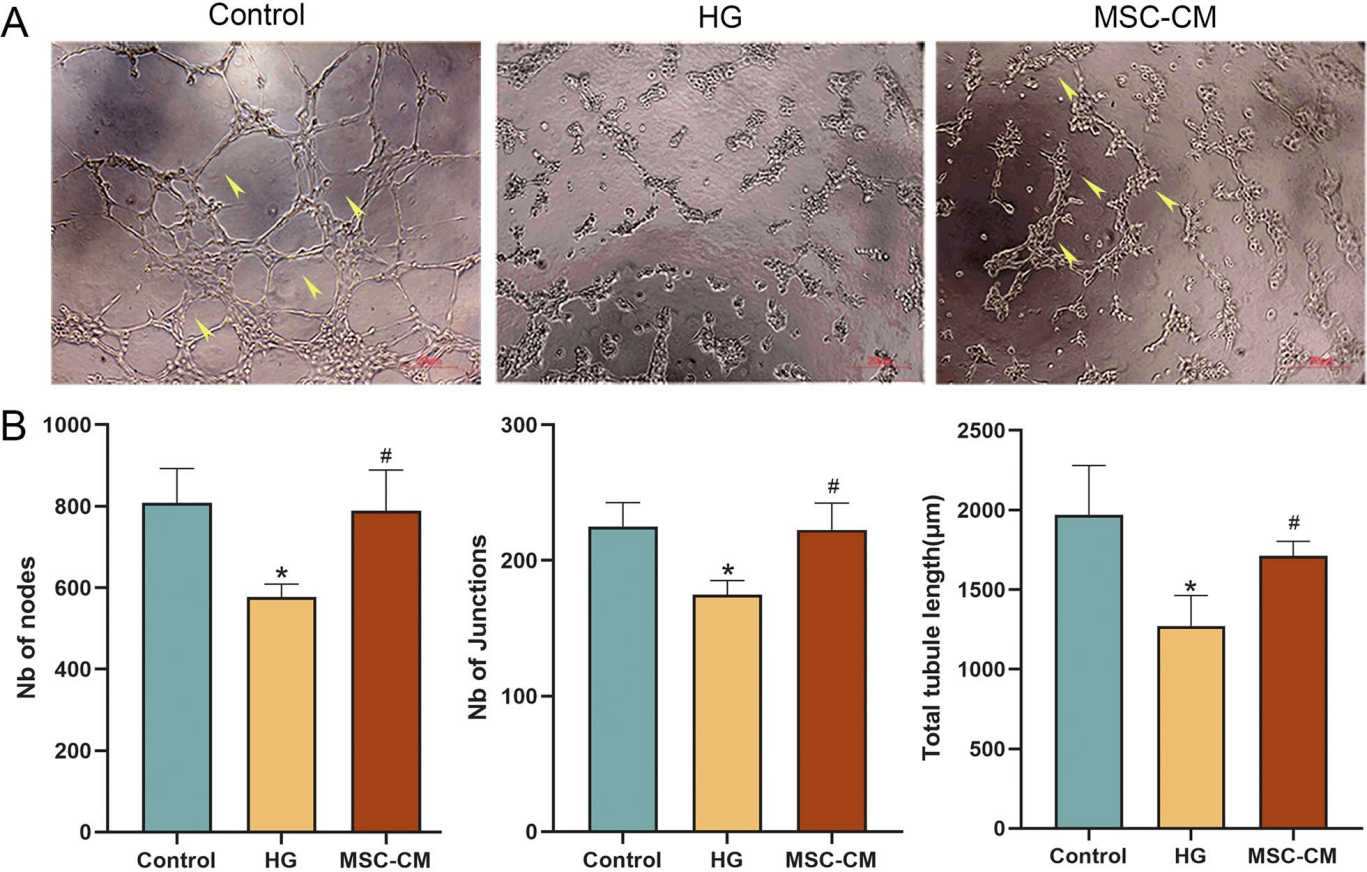
The effect of hucMSCs on tubule formation of HUVECs. **A** Tube networks formation of HUVECs in the control, HG and MSC-CM groups (scale bar = 20 μm). **B** Nb nodes, Nb junctions, and total tubule length of tube networks in the control, HG and MSC-CM groups. Data were mean ± SD, *P < 0.05 versus control level, and ^#^*P* < 0.05 versus model level

Matrigel tube formation assay was applied to evaluate the effect of MSC-CM on the tube formation ability of HUVECs. As shown in Fig. 5A, compared with the control group, the tube formation of HUVECs was severely disrupted, on which MSC-CM exerted a curative effect. In Fig. 5B, the statistical analysis of tube networks on number of junctions (Nb nodes), number of junctions (Nb junctions), and total tubule length of tube networks were all significantly deceased in the model group (each *P* < 0.05 vs control level) and restored in the MSC-CM group (each *P* < 0.05 vs model level).

### Molecular regulation of MSC-CM on diabetic HUVECs

**Fig. 6:**
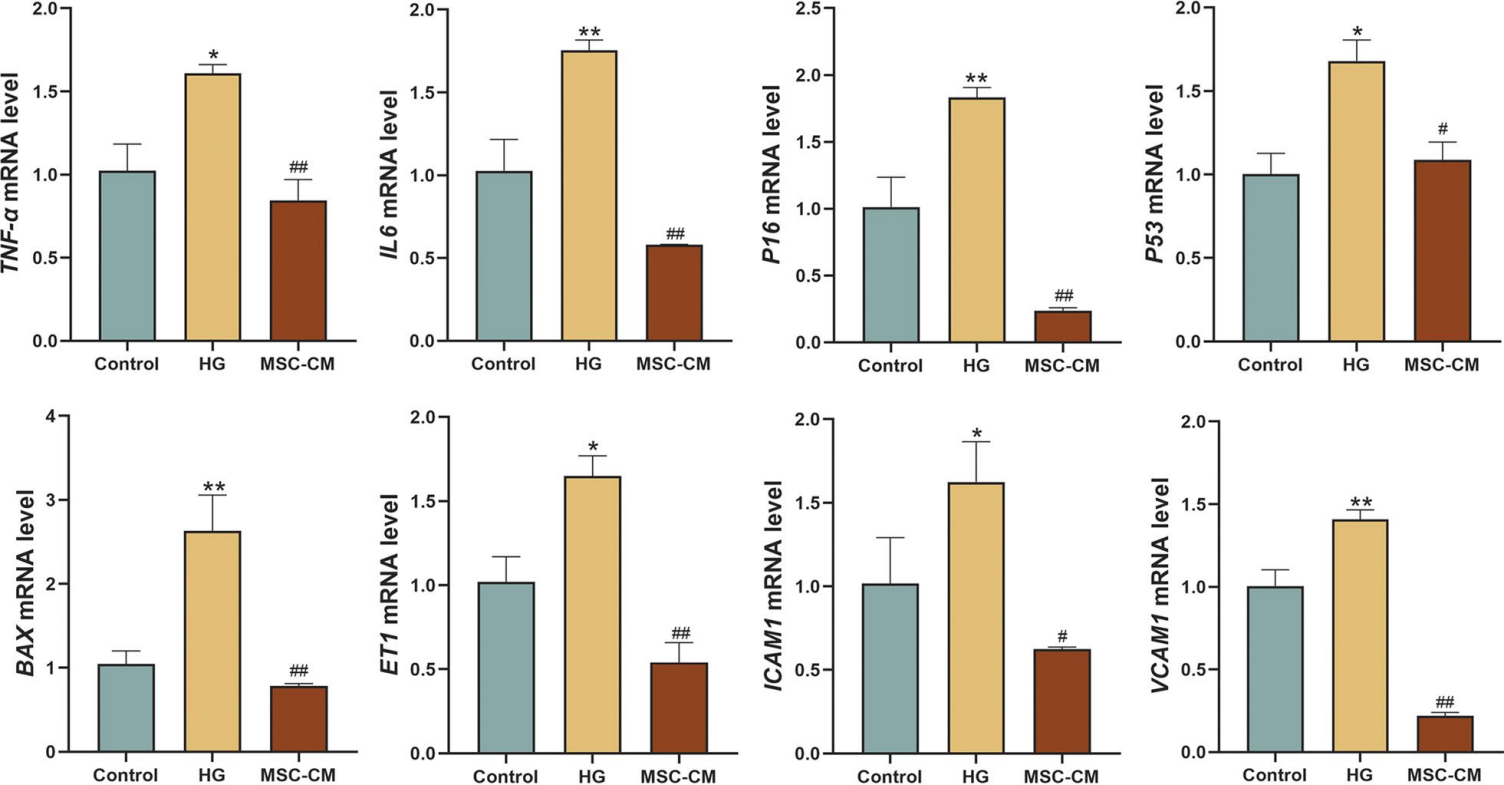
mRNA expressions cell senescence and tube formation related genes of HUVECs in the control, HG and MSC-CM groups. Data were mean ± SD, *P < 0.05 or **P < 0.01 versus control level, and ^#^*P* < 0.05 or ^##^*P* < 0.01 versus model level

The mRNA expressions of cell senescence (*P16*, *P53*, *BAX*, *IL-6*, and *TNF-α*) and tube formation (*ET1*, *ICAM-1*, and *VCAM-1*) related genes were tested to analyze the molecular regulation of hucMSCs on HUVECs in high glucose condition. As shown in Fig. 6, high glucose significantly up-regulated the mRNA expressions of those genes (each *P* < 0.05 or *P* < 0.01 vs control level), while MSC-CM effectively restored the alterations (each *P* < 0.05 or *P* < 0.01 vs model level), indicating positive molecular actions of hucMSCs on cell senescence and tube formation suppression of diabetic HUVECs.

### Paracrine effects of hucMSCs on HUVECs viability, migration and mRNA expression in co-culture way

**Fig. 7:**
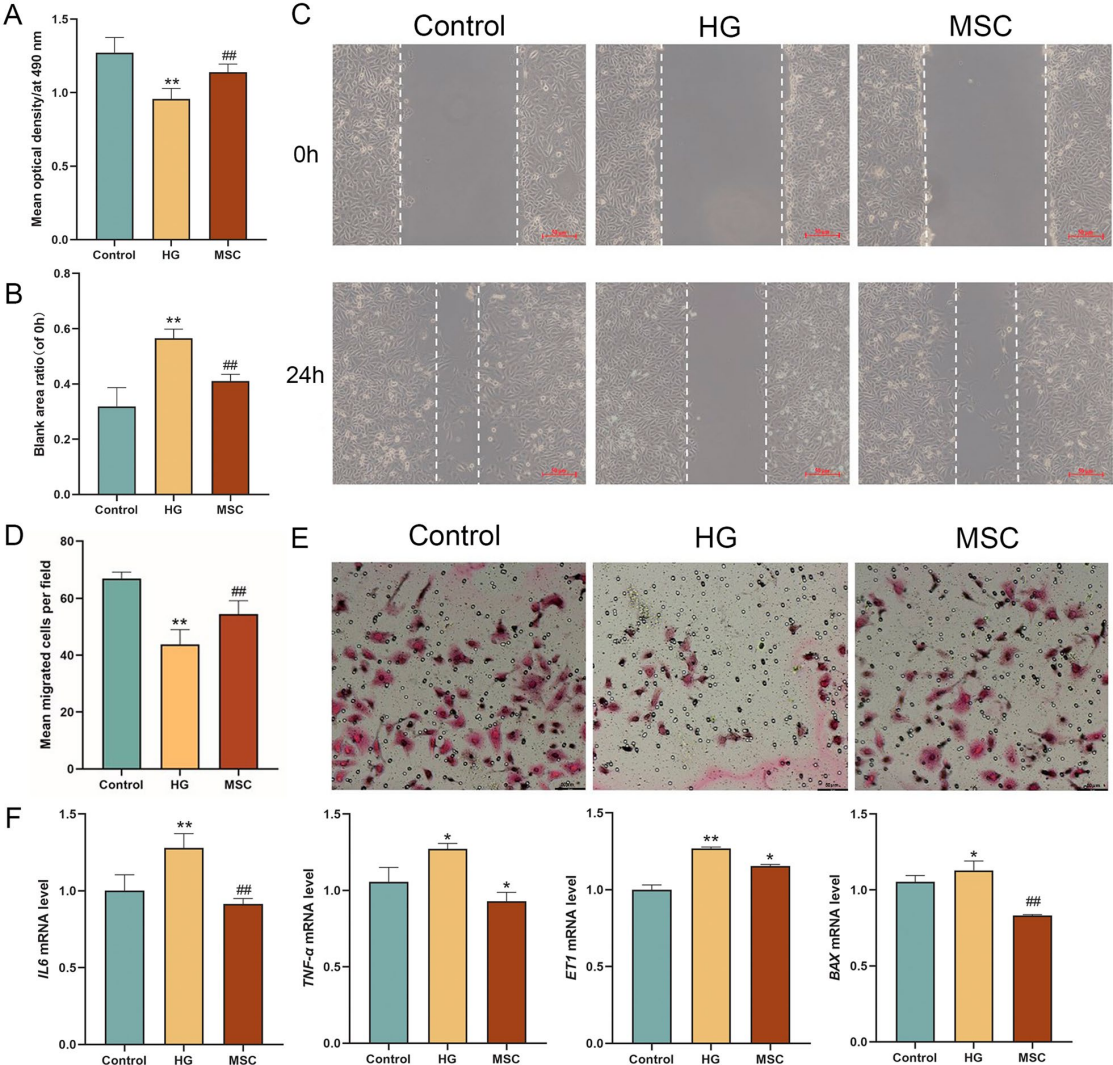
Effects of hucMSCs on cell viability, wound healing, cell migration and mRNA expression of HUVECs under co-culture conditions. **A** Cell viability was detected by MTT method. **B**, **C** wound healing rate and representative pictures (scale = 50 μm). **D**, **E** showed the number of cell migration and the representative images in the transwell experiment. Four regions were randomly selected for cell counting in each group (scale = 50 μm). **F** mRNA expression in inflammation, migration and apoptosis-related genes

To confirm the paracrine effects of hucMSCs on HUVECs, cell viability assay, wound healing transwell assay and PCR were conducted under co-culture condition. In Fig. 7A, the cell viability in the HG group was significantly lower than that in the control group (*P* < 0.01 vs control level), while that in the MSC group was significantly improved (*P* < 0.01 vs model level). In Fig. 7B, C, the wound healing ability of HUVECs in the HG group was significantly inhibited (*P* < 0.01 vs control level), while that in the MSC group was restored to normal (*P* < 0.01 vs model level). In Fig. 7D, E, the transwell migration of HUVECs in the HG group was significantly inhibited (*P* < 0.01 vs control level), while that in the MSC group was significantly restored to normal (*P* < 0.01 vs model level). In Fig. 7F, the mRNA expression levels of inflammation (*IL6*, *TNF-α*), migration (*ET1*), and apoptosis (*BAX*) in the HG group were significantly up-regulated (each *P* < 0.01 vs control level), while those in the MSC group were significantly reversed (each *P* < 0.01 vs model level). The above results had similar tendency with that of MSC-CM treatment.

### RNA-seq and KEGG enrichment analysis

**Fig. 8:**
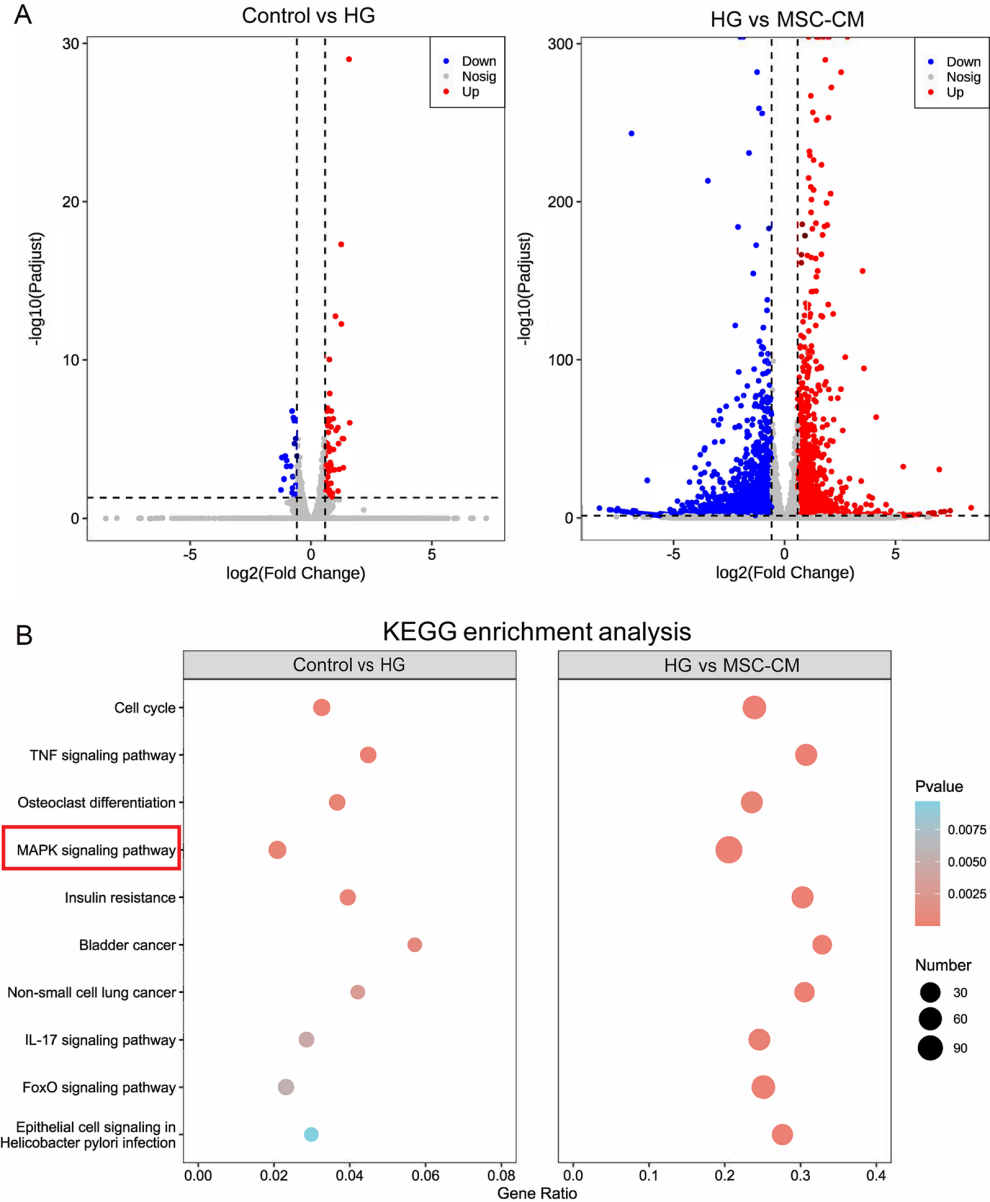
RNA-seq analysis of differentially expressed genes (DEGs) and Kyoto encyclopedia of genes and genomes (KEGG) pathways in the control versus HG and HG versus MSC-CM groups. **A** volcano plot profile, and **B** significantly enriched KEGG signaling pathways

To explore the mechanism by which MSC-CM protected HUVECs from diabetic damage, RNA-seq and KEGG enrichment analysis were conducted in control vs HG and HG vs MSC-CM groups. About 50 to 85 million raw reads were obtained from the control, HG and MSC-CM groups, and nearly 49 to 84 million clean reads were attained. More than 94% of the clean reads had a high-quality score ≥ Q30 level (sequencing error rate < 0.02%), supporting the preciseness of sequencing. Figure 8A shows the volcano plot result that a total of 97 and 1886 DEGs were identified in control vs HG and HG vs MSC-CM groups, respectively. In Fig. 8B, significantly enriched KEGG signaling pathways were obtained according to the gene number and *P* value < 0.05, including cell cycle, TNF signaling pathways, osteoclast differentiation, MAPK signaling pathway, and insulin resistance, etc. MAPK/ERK signaling pathway was selected for the mechanism study.

### Verification of RNA-seq-based mechanism of huMSCs on MAPK signaling pathway

**Fig. 9:**
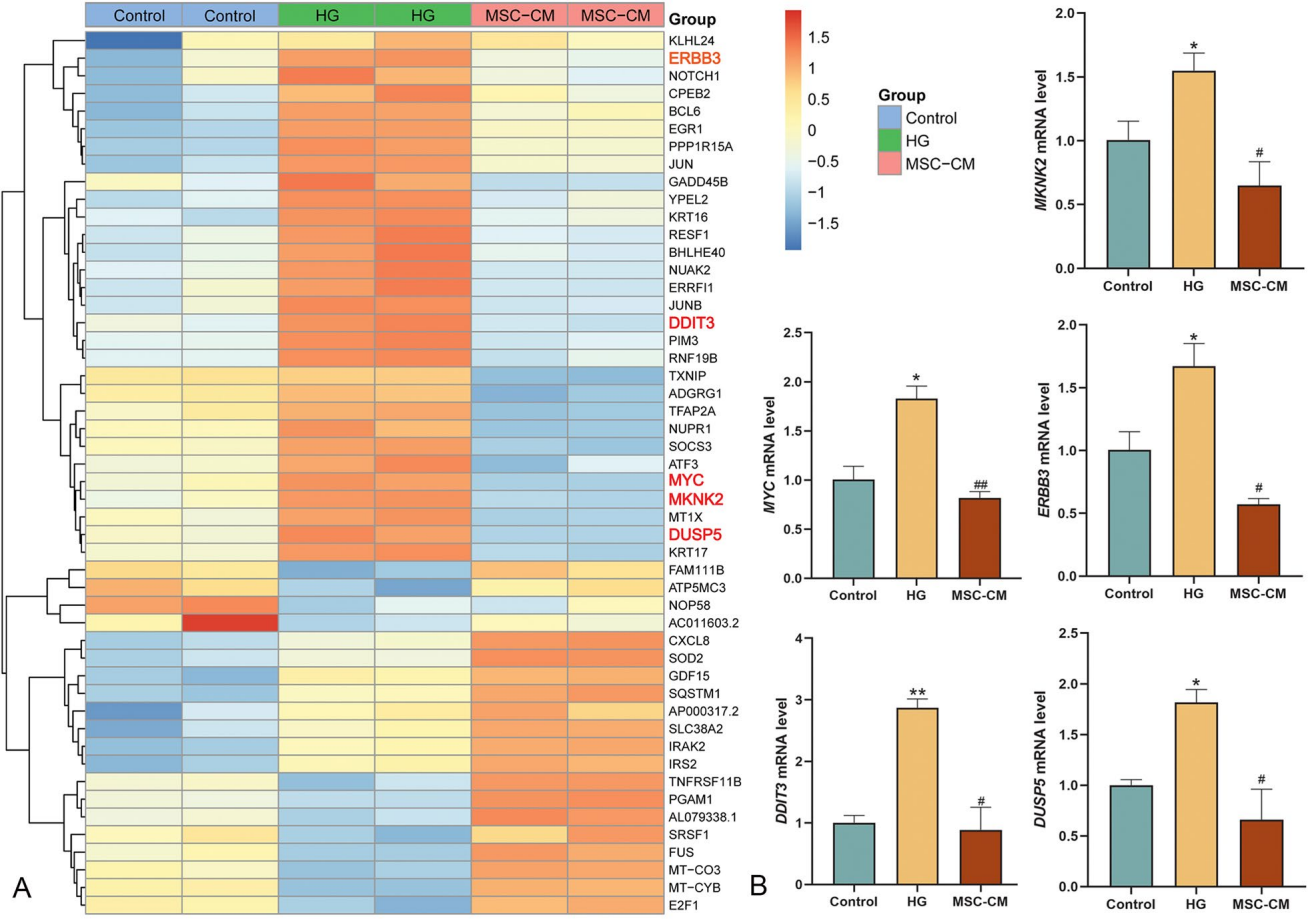
Identification and verification of hub differentially expressed genes (DEGs) in the control, HG and MSC-CM groups. **A** Heatmap showed the expression levels of top 50 DEGs in different groups. **B** Verification of key genes (*FERB3*, *DDIT3*, *MYC*, *MKNK2* and *DUSP5*) using qPCR. Data showed mean ± SD, *P < 0.05 and **P < 0.01 compared to the control group, ^#^*P* < 0.05 and ^##^*P* < 0.01 compared to the model group

To validate the RNA-seq data, the MAPK/ERK signaling pathway-related genes were analyzed by qPCR. In Fig. 9A, the heatmap showed top 50 of the significantly changed DEGs, among them, five representative genes (*FERB3*, *DDIT3*, *MYC*, *MKNK2* and *DUSP5*) in the MAPK signaling pathway were picked. As shown in Fig. 9B, these genes were significantly up-regulated in the model group (each *P* < 0.05 or *P* < 0.01 vs control level) and were significantly restored by MSC-CM (each *P* < 0.05 or *P* < 0.01 vs model level), indicating a same tendency with RNA-seq data (Table 2).

**Table 2:** Differentially expressed genes in the control versus HG and HG versus MSC-CM groups

Gene	Control versus HG		HG versus MSC-CM		KEGG analysis
	log2 (fold change)	*P* value	log2 (fold change)	*P* value	
*ERBB3*	1.498725752	1.40318E−07	1.675840782	1.75399E−32	MAPK/ERK
*DDIT3*	2.385524224	1.8222E−16	1.331057428	1.08703E−54	MAPK/P38
*MYC*	1.41915689	4.94648E−07	1.494554545	7.85089E−76	MAPK/ERK
*MKNK2*	1.329319884	1.74241E−05	1.580058046	7.09772E−72	MAPK/ERK
*DUSP5*	1.653715501	5.93044E−10	1.409875214	4.76505E−80	MAPK/ERK

### Protein regulation of hucMSCs on diabetic HUVECs and thoracic aorta.

**Fig. 10:**
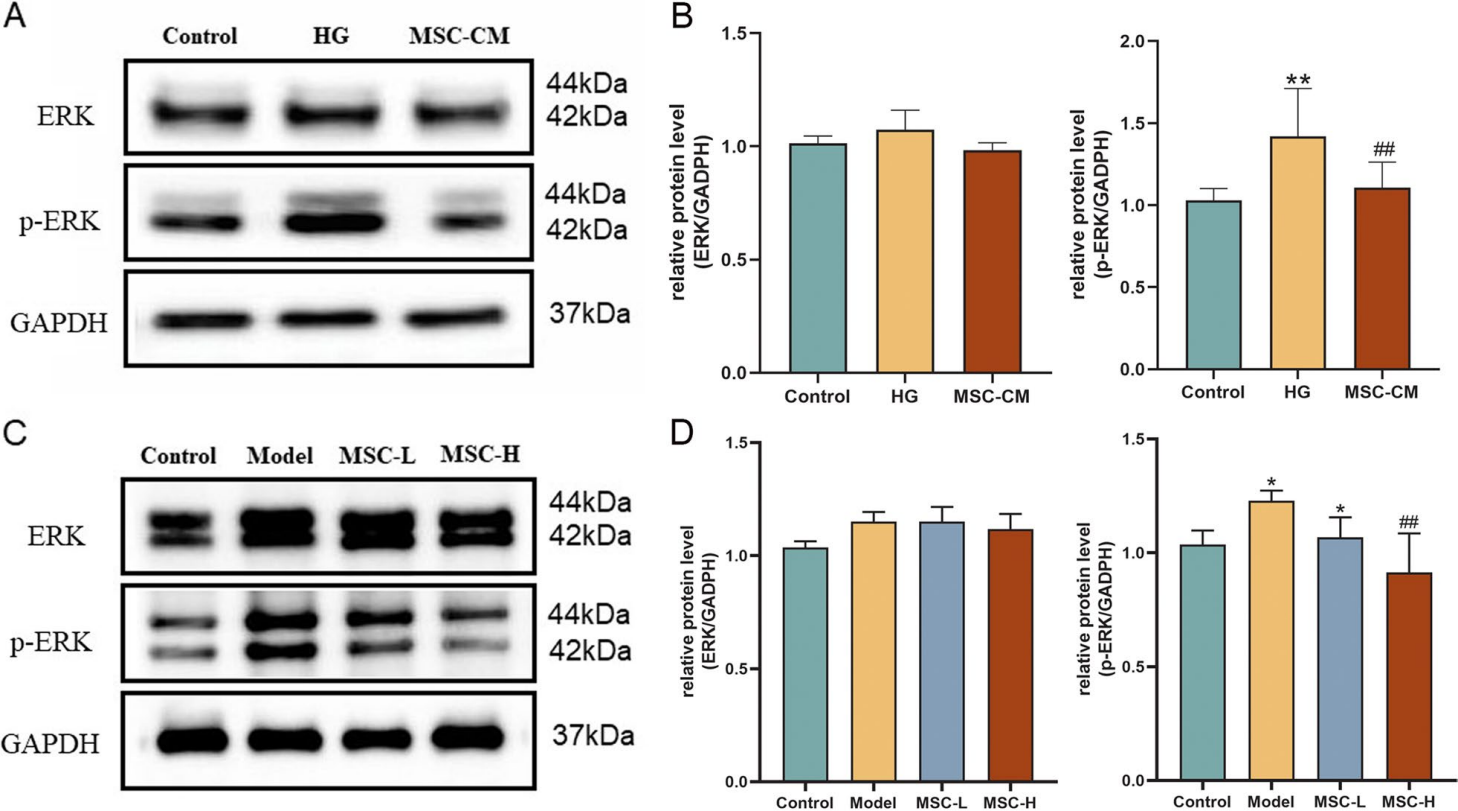
Western blot of protein expressions related to the MAPK/ERK signaling pathway in HUVECs and thoracic aorta. **A** Representative images of ERK, p-ERK and GADPH in HUVECs; **B** Histogram of statistical analysis on ERK and p-ERK protein levels in HUVECs; **C** Representative images of ERK, p-ERK and GADPH in thoracic aorta; and **D** Histogram of statistical analysis on ERK and p-ERK protein levels in thoracic aorta. For statistical analysis of the protein expression, Image-J software (Version 1.49, National Institutes of Health, Bethesda, USA) was applied to quantify the gray value of each protein blot, and the ratio of gray value of the target blot (ERK and p-ERK) to that of the internal reference blot (GADPH) was calculated as the relative protein level in the histogram. All data were repeated for three times and added in supplementary data. Data was mean ± SD, *P < 0.05 and **P < 0.01 versus control level, ^#^*P* < 0.05 and ^##^*P* < 0.01 versus model level

To further explore the molecular mechanism of hucMSC on the MAPK/ERK signaling pathway, the protein expressions of ERK and phosphorylated ERK (p-ERK) in vitro and in vivo were detected using WB analysis. As shown in Fig. 10A, B, in vitro results indicated that no significant changes of ERK protein were found in each group, moreover, phosphorylated ERK was significantly up-regulated in the model group (*P* < 0.01 vs control level) and was significantly restored by MSC-CM (*P* < 0.01 vs model level). In Fig. 10C, D, the in vivo results also showed similar tendency with the in vitro data, in which the expression of p-ERK was significantly up-regulated in the model group (*P* < 0.05 vs control level), while that in the MSC-L or MSC-H groups was significantly reversed (*P* < 0.05 or *P* < 0.01 vs model level). Moreover, both in vivo (samples from three individual rats) and in vitro (samples from HUVECs in three protein extractions), the similar results and trends as described above were successfully repeated for three times and presented in the Additional file 1: Fig. S2 and S3, which confirmed that WB results were robust and reproducible in this study.

## Discussion

**Fig. 11:**
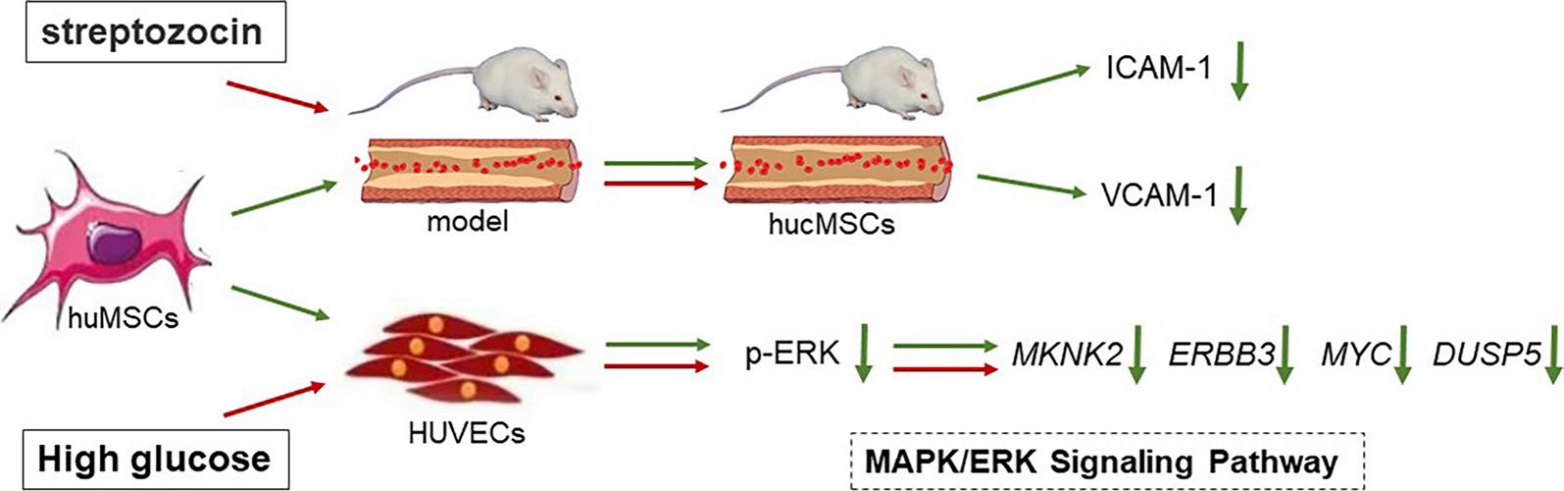
Mechanism of hucMSCs on modifying negative impact of glucose on endothelial cell injury. The red arrows represent the high glucose-induced injury process and the green arrows represent the MSCs intervention process

Diabetic vascular complications are most common cause of death in people with diabetes mellitus, resulting in macrovascular (atherosclerosis and heart disease) and microvascular (nephropathy, retinopathy and neuropathy) diseases [2]. Along with the increasing incidence of diabetes mellitus worldwide, a heightened risk of vascular disease comes, which in turn leads to the explosive mortality of diabetic patients and a medical burden to society [2, 3]. Endothelial damage or dysfunction plays an initial role in the process of diabetic vascular complications, characterized by increased secretion of vasoconstriction factors, damaged endothelial cells, disordered endothelial permeability, and disordered thrombosis, etc. [4, 27]. For example, endothelial cells were severely damaged in the early stage of diabetic retinopathy, caused by chronic inflammation, leukotasis, oxidative stress, and dysregulated cytokines, further resulting in loss of pericytes, form of acellular capillaries from retinal capillaries, increase of vascular permeability, and break down of inner blood-retinal barrier [28]. Besides, diabetes-induced endothelial damage could trigger nephropathy by inducing nodular glomerulosclerosis, glomerular basement membrane thickness, and mesangial expansion, ultimately declining glomerular filtration rate [29]. Moreover, endothelial cell senescence plays a key role in the development of diabetic cardiovascular diseases, e.g., atherosclerosis, exhibiting features of enlarged cell appearance, enhanced inflammatory secretion and SA-β-gal activity, and increased expressions of aging-related genes and proteins [30]. Therefore, endothelial damage can be regarded as an important target for the treatment of diabetic vascular complications, which has been focused by this study. Our data indicated that hucMSCs not only ameliorated blood glucose, but also repaired the diabetic damage of endothelium by improving the cell viability, wound healing capability, migration, senescent and inflammatory state, and angiogenesis of HUVECs, in which MAPK/ERK signaling mediated its molecular mechanism of paracrine action (Fig. 11). The innovative highlights of this study were as follows: 1) first report on the role of MSCs in repairing diabetic vascular damage; 2) reveal of the paracrine action mode of hucMSCs on the damaged endothelial cells; and 3) discovery of MAPK/ERK signaling pathway-mediated mechanism of hucMSCs on repairing endothelium.

Diabetic vascular endothelial damage usually occurs with cell senescence, oxidative stress, inflammation, mitochondrial apoptosis, and disordered thrombosis [4], providing potential clues for development of therapeutic strategy and for mechanism study of therapeutics. However, the effect of MSCs on diabetic complications, especially the vascular complications, is rarely reported. Previously, MSCs have been widely studied for treatment of diabetes mellitus, and their effect of blood glucose regulation has been evaluated [31]. For instance, Zhu et al. discovered that repeated infusion of bone marrow MSCs had only a slight effect of blood glucose regulation in type I diabetic rats [32]. In this study, similar outcome was obtained that 4-week injection of hucMSCs partially decreased blood glucose level of type I diabetic rats. The weak effect might be possibly due to the thorough damage of pancreatic β-cells by STZ, resulting in difficulty of MSCs in complete repair of damaged β-cells or supplement of new β-cells. Despite hucMSCs resulted in slight improvement in body weight and fasting blood sugar level, the vascular endothelial restoration was more significant. Hence, the innovative point of our finding was the determination of hucMSCs′s efficacy and mechanism on diabetic damage of vascular endothelium, and its hypoglycemic effect was incidentally verified. To determine whether endothelium was damaged by high blood glucose in the STZ model, in vitro model of HUVECs was established and high glucose (50 mM glucose) culture was applied. The in vitro results showed that high glucose caused HUVECs damage by decreasing cell viability, wound healing, migration, and angiogenesis and increasing senescent and inflammatory state. In comparison, STZ treatment on HUVECs caused little change of inflammation (*TNF-α* and *IL6*), migration (*ET-1*), and apoptosis (*BAX*) parameters (each *P* > 0.05 vs normal levels) (Additional file 1: Fig. S1). The selected dose of STZ (2 mmol/l) has been reported to substantially damage islet β cells [33]. Accordingly, the direct damage of vascular endothelium in diabetic rats was mainly due to high blood glucose other than STZ, and thereby the HUVECs model was rationally used. In sum, since hucMSCs exerted vascular protective effects more significantly than its effect on blood sugar level and body weight, a proposition was posed that MSCs can be used as an adjuvant for type I diabetes therapy, but not an alternative option to replace current anti-diabetic drugs. Paracrine is known as a main mode of action of MSCs, which plays therapeutic roles in treating endothelial damages associated with oxidative stress, inflammation, mitochondrial apoptosis, and cell senescence [16]. Previous study reported that the paracrine product of bone marrow-derived mesenchymal stem cells (MSC-CM) attenuated the oxidative stress and apoptosis of endothelial cells through Sirt1/AMPK/PGC-1α pathway [34]. Besides, bone marrow-derived mesenchymal stem cells (bMSCs) were found to release trophic factors (VEGF-1α, ANG, HIF-1α and MMP-9) and increase expressions of VEGF-1α, MMP-9, pAKT and VEGF-R in HUVECs to activate VEGF/AKT signaling pathway, which promoted the proliferation, inhibited the apoptosis, and increased the migratory and tube forming capacity of HUVECs [35]. VEGF was found as an important paracrine factor of MSCs, and the VEGF overexpressed conditional medium (MSC-VEGF-CM) promoted the survival and migration of microvascular endothelial cells in pancreatic islet through reactivation of PI-3 K/AKT/m-TOR/eNOS and p38/MAPK signaling pathways [36]. Therefore, MSCs in paracrine mode should be effective in treating diabetes-induced vascular endothelial damage, which was evidenced by this study that MSC-CM from hucMSCs significantly attenuated high glucose-induced cell senescence and promoted cell viability, wound healing, migration and angiogenesis of HUVECs through MAPK/ERK signaling pathway.

MAPK (Mitogen-activated protein kinases) family contains ERK (extracellular signal-regulation kinase), JNK (c-jun-N-terminal kinase) and p38 kinase [37]. MAPK/ERK pathway is associated with cell proliferation, differentiation, migration, senescence and apoptosis [38]. In this study, high glucose activated MAPK/ERK signaling pathway in HUVECs through overexpression of *MKNK2*, *ERBB3*, *MYC* and *DUSP5* and phosphorylation of ERK, whereas MSC-CM protected HUVECs from high glucose through inhibition of this pathway, partially explaining how hucMSCs treat diabetic vascular complications. Of these molecules, *MKNK2* is a substrate of MAPK/ERK pathway and its isoform Mnk2a directly activates and translocates p38-MAPK into the nucleus, leading to the activation of its target genes and cell death [39]. It can thereby be speculated that MSC-CM reduced the expression of *MKNK2* to inhibit apoptosis of HUVECs. As a regulator of cytoskeletal dynamics in microvascular endothelial cells, *ERBB3* affects vascular endothelial permeability and tight junction levels [40], indicating that MSC-CM ameliorated the damaged vascular endothelial permeability with diabetes by regulation of *ERBB3*. *MYC* induces selective splicing of signal kinase *MKNK2* and is a downstream molecule of ERK signaling that regulates endothelial cell proliferation and migration, inhibition of which can promote the migration of HUVECs [41]. *MYC* is related to transcriptional activity, and its influence on ERK also affects the transcription of *DUSP5* [42]. Studies have shown that *ERBB3* regulates AKT2 phosphorylation to further activate transcription factor *MYC* [43]. Moreover, *DUSP5*, with considerable nuclear anchoring activity, functions to dephosphorylate ERK1/2 to inhibit proliferation of endothelial cells [44]. Hence, down-regulation of *MYC* and *DUSP5* by the treatment of MSC-CM might contribute to the proliferation and migration of the high glucose-damaged HUVECs. In the immunohistochemical analysis of aortic tissue samples, we also found similar tendency of ERK1/2 phosphorylation with the in vitro data in HUVECs, accompanying with positive regulation of HH3. HH3 plays a crucial role in coagulation and thrombosis, which induces overexpression of adhesion molecules (ICAM-1 and VCAM-1) and procoagulant molecule tissue factor, resulting in inflammatory cell recruitment and thrombosis [45]. The results indicated that hucMSCs exerted positive effects on aortic tissues with regulations of ERK1/2 and histone H3, which partially verified the in vitro mechanism study of hucMSCs in the HUVEC system.

Previous studies have shown that transplanted MSCs usually have low survival rate in target tissues and their paracrine capacity is considered to play a crucial role in repairing tissues [46, 47]. In this study, a paracrine action mode of hucMSCs has been demonstrated, but the material basis in MSC-CM remains unclear. To date, a series of paracrine molecules excreted by MSCs have been discovered, including cytokines, growth factors, exosomes, and non-coding RNAs. Many of them are involved in the regulation of MAPK/ERK signaling pathway (Table 3) [45–62]. Of these, ERBB4, Spred1, miR-125b, HOTAIRM1-1, and miR-133a are negative regulators, while HIF-2α, IGF-1, TGF-β3, bFGF, VEGF, BDNF, METTL1, FGF8, IGF-1, SCGF-beta, HGF, MCP-1, miR-126, SMSCs-126-Exos, ApoE, IL-8, and IL-6 are positive regulators. These molecules may play important roles in the action mechanism of hucMSCs on diabetic vascular complications, warranting further investigation.

**Table 3:** Chemical factors from stem cells on MAPK/ERK signaling pathway

Chemical factors	Cell source	Function (MAPK/ERK)	References
ERBB4	Mesenchymal stem cells	Inhibition	[48]
Spred1	Neural stem cells	Inhibition	[49]
miR-125b	Mesenchymal stem cells	Inhibition	[50]
HOTAIRM1-1	Mesenchymal stem cells	Inhibition	[50]
miR-133a	Mesenchymal stem cells	Inhibition	[51]
HIF-2α	Mesenchymal stem cells	Activation	[52]
IGF-1	Mesenchymal stem cells	Activation	[53]
TGF-β3	Mesenchymal stem cells	Activation	[53]
bFGF	Neural stem cells	Activation	[54]
VEGF	Multi-potent progenitor cells	Activation	[55]
BDNF	Mesenchymal stem cells	Activation	[56]
METTL1	Embryonic stem cells	Activation	[57]
FGF8	Spermatogonial stem cells	Activation	[58]
IGF-1	Neural stem cells	Activation	[59]
SCGF-beta	Mesenchymal stem cells	Activation	[60]
HGF	Mesenchymal stem cells	Activation	[60]
MCP-1	Mesenchymal stem cells	Activation	[60]
miR-126	Mesenchymal stem cells	Activation	[61]
SMSCs-126-Exos	Mesenchymal stem cells	Activation	[62]
ApoE	Neural stem cells	Activation	[63]
IL-8	Mesenchymal stem cells	Activation	[64]
IL-6	Mesenchymal stem cells	Activation	[65]

## Conclusion

This is the first study reporting hucMSCsʹ efficacy not only on diabetes but also on diabetic vascular endothelial damage. The in vivo data demonstrated that hucMSCs ameliorated the high blood glucose and body weight of diabetic rats and also repaired the damaged vascular endothelium in aspects of histopathology and vascular function. The in vitro data further confirmed that hucMSCs improved cell viability, wound healing, migration, angiogenesis, and cell senescent state of the high glucose-damaged HUVECs through a paracrine action mode. Moreover, the paracrine mechanism of hucMSCs was clarified to be mediated by MAPK/ERK signaling pathway. Further studies are required to explore what paracrine molecules are mainly responsible for the efficacy of hucMSCs. In sum, our findings provided novel knowledge of hucMSCs in the treatment of diabetes and suggested a prospective strategy for the clinical treatment of diabetic vascular complications.

## Supplementary Information

**Additional file 1**.** 1.** The mRNA expressions of STZ treatment on HUVECs;** 2.** Repeated results of protein regulation in HUVEC and thoracic aorta.

## Data Availability

The datasets supporting the conclusions of this article are available in the SRA database, with unique accession code PRJNA774479 and hyperlink to dataset(s) in https://www.ncbi.nlm.nih.gov/Traces/study/?acc=PRJNA774479. All other data are concluded in this article.

## Abbreviations

MSCs: Mesenchymal stem cells
hucMSCs: Human umbilical cord-derived MSCs
FBG: Fasting blood glucose
RNAseq: RNA sequencing
MSC-CM: Conditioned medium of hucMSCs
ROS: Reactive oxygen species
ET-1: Endothelin-1
NO: Nitric oxide
Ang II: Angiotensin II
p-ERK: Phosphorylated ERK
STZ: Streptozotocin
HH3: Histone H3
BMSCs: Bone marrow-derived mesenchymal stem cells
MAPK: Mitogen-activated protein kinases
ERK: Extracellular signal-regulation kinase
JNK: C-jun-N-terminal kinase
