# Supplementary material for: Human umbilical cord-derived mesenchymal stem cells not only ameliorate blood glucose but also protect vascular endothelium from diabetic damage through a paracrine mechanism mediated by MAPK/ERK signaling
Source: Stem Cell Res Ther. 2022 Jun 17;13:258. doi: 10.1186/s13287-022-02927-8 (PMC9205155; doi:10.1186/s13287-022-02927-8)
Supplement: Supplementary file 1 — Additional file 1. 1. The mRNA expressions of STZ treatment on HUVECs; 2. Repeated results of protein regulation in HUVEC and thoracic aorta. [file 13287_2022_2927_MOESM1_ESM.docx]

1. The previous literatures were analyzed and it was found that STZ at the concentration of 2 mmol/L will damage islet β cells. The control group, high glucose (HG) group and STZ group were established. After 48 h treatment, RNA was extracted with the same method as in the manuscript, and qPCR was performed. The results showed that the mRNA expressions of inflammation-related (*TNF-α* and *IL6*), migration-related (*ET-1*) and apoptosis-related (*BAX*) genes in the HG group were significantly up-regulated compared with the control group, while those in the STZ group were not significantly changed compared with the control group, which confirmed that STZ did not cause significant damage to HUVECs.


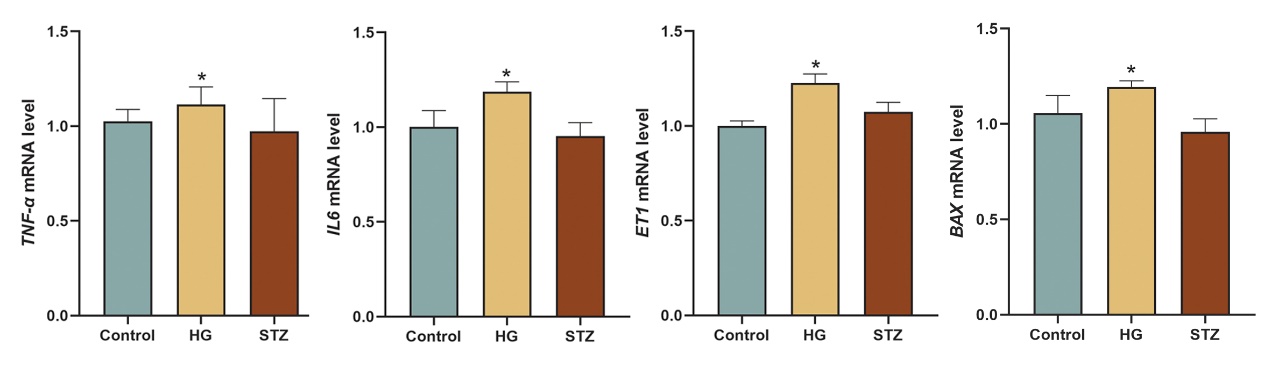


**Figure S1.** The mRNA expressions (*TNF-α*, *IL6,* *ET-1*and *BAX*) of HUVECs in the control, HG, and STZ groups were detected by qPCR. Data were mean ± SD. ^*^*P* < 0.05 and ^**^*P* < 0.01 vs control.

1. In order to ensure the reproducible of WB results, three repeated experiments of ERK and p-ERK protein detection were conducted and quantitatively analyzed. As shown in Figure S2, three repeated in vitro results indicated that no significant changes of ERK protein were found in each group, moreover, phosphorylated ERK was significantly up-regulated in the model group (P < 0.01 vs control level) and was significantly restored by MSC-CM (P < 0.01 vs model level). In Figure S3, three repeated in vivo results also showed similar tendency with the in vitro data, in which the expression of p-ERK was significantly up-regulated in the model group (P < 0.05 vs control level ), while that in the MSC-L or MSC-H groups was significantly reversed (P < 0.05 or P < 0.01 vs model level). Obviously, in vitro and in vivo, three repeated WB experiments above were robust and reproducible.


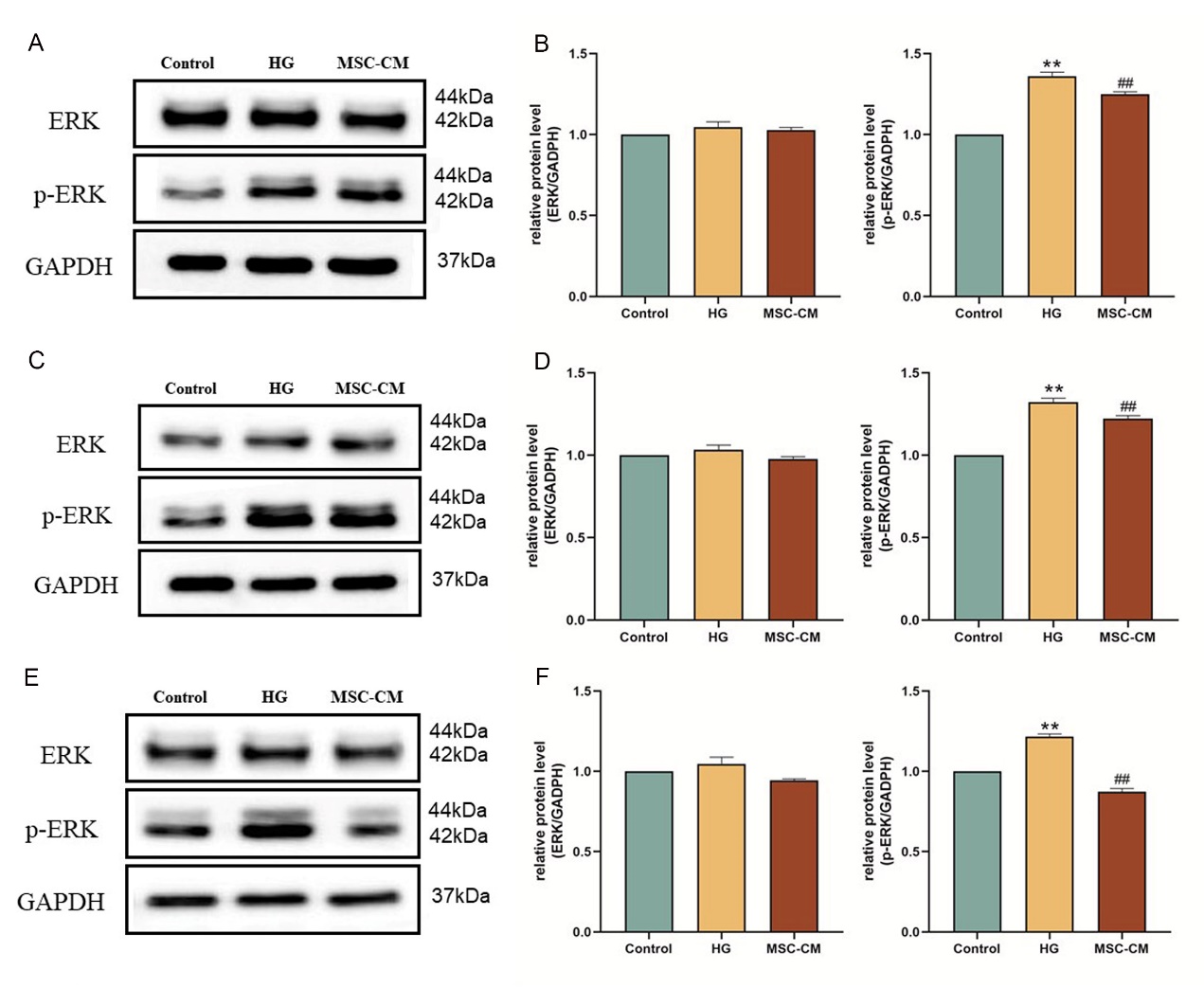


**Figure S2：**Repeated results of protein regulation in HUVECs. (A) (C) and (E) Representative images of ERK, p-ERK and GADPH in HUVECs; (B) (D) and (F) Histogram of statistical analysis on ERK and p-ERK protein levels in HUVECs. Data was mean ± SD, **P* < 0.05 vs control level, ^#^*P* < 0.05 vs model level.


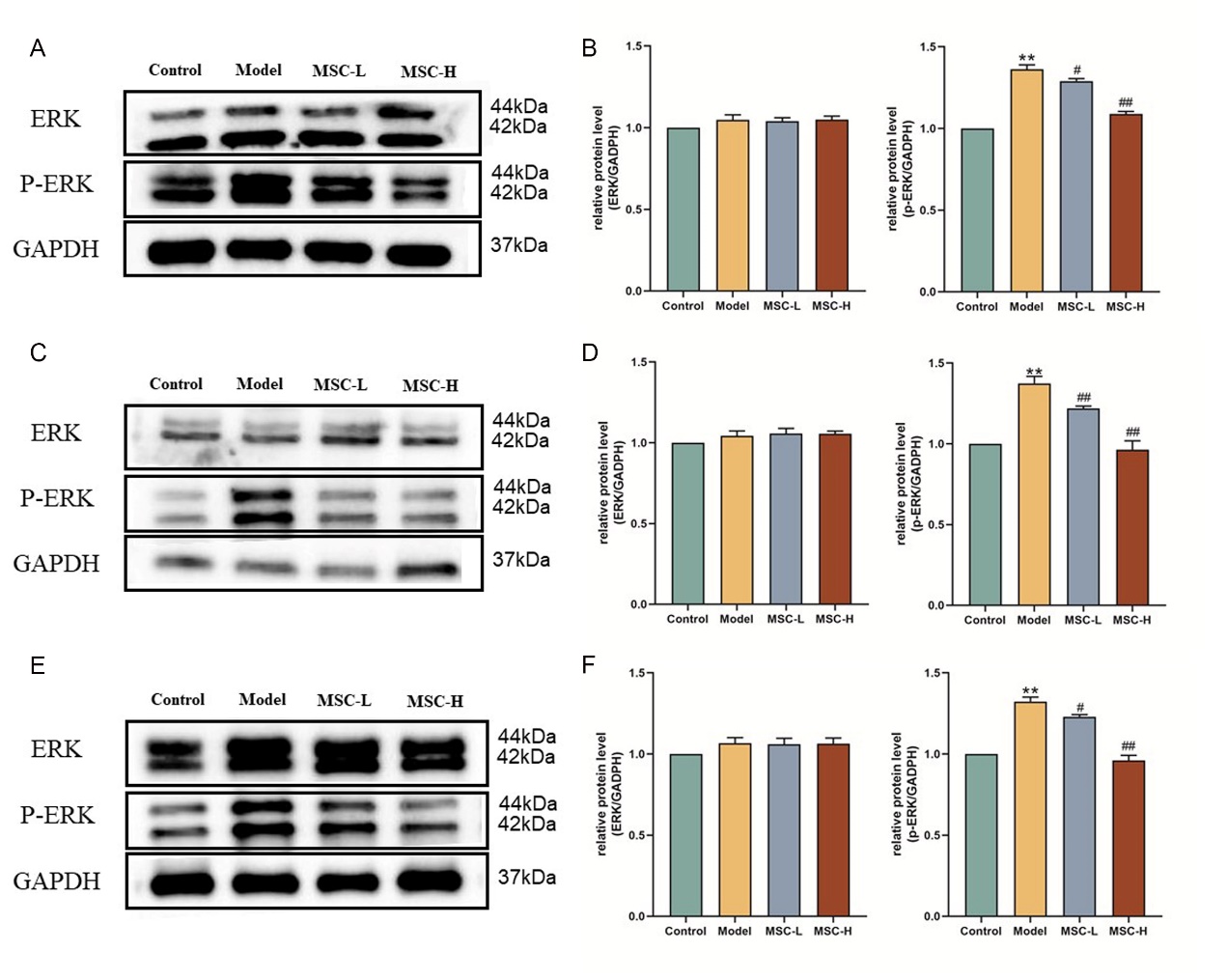


**Figure S3：**Repeated results of protein regulation in thoracic aorta. (A) (C) and (E) Representative images of ERK, p-ERK and GADPH in thoracic aorta; (B) (D) and (F) Histogram of statistical analysis on ERK and p-ERK protein levels in thoracic aorta. Data was mean ± SD, ***P* < 0.01 vs control level, ^#^*P* < 0.05 and ^##^*P* < 0.01 vs model level.
